# Child centred policing: supporting trauma informed front line police practices with girls who have lived experiences of child sexual exploitation in the United Kingdom

**DOI:** 10.3389/fpubh.2026.1697743

**Published:** 2026-02-10

**Authors:** Tracee Green, Aravinda Kosaraju, Emma Soutar

**Affiliations:** Centre for Child Protection, School of Social Sciences, University of Kent, Gillingham, United Kingdom

**Keywords:** child sexual exploitation (CSE), evaluation, policing, simulation, training, trauma-informed approach, violence against women and girls (VAWG)

## Abstract

**Introduction:**

Child sexual exploitation (CSE) continues to present significant challenges for policing, with longstanding concerns about inconsistent recognition of risk, victim-blaming narratives, and uneven application of trauma-informed approaches.

**Methods:**

This study reports on the co-design, delivery, and evaluation of a pilot simulation-based training tool developed by the University of Kent’s Centre for Child Protection in partnership with Kent Police. Guided by a participatory action research framework, a mixed-methods evaluation was conducted, including pre-, mid-, and post-training questionnaires, live polling, and independent observation.

**Results:**

Quantitative findings demonstrated improvements in officers’ self-reported confidence, knowledge, and practice skills. Qualitative feedback indicated reductions in victim-blaming language and greater use of trauma-informed framing. Observations reinforced these shifts, highlighting enhanced officer engagement with contextual safeguarding and recognition of trauma responses.

**Discussion:**

Although limited to a single police force and reliant on self-reported data, the pilot suggests that simulation-based learning is a promising approach to embedding trauma-informed practice in frontline policing. The findings underline the value of experiential training for disrupting entrenched biases and supporting culture change in sensitive areas of practice.

## 1 Introduction

National and global polices have turned attention towards tackling violence against women and girls (VAWG), targeting any act of violence against females. The World Health Organisation (1) estimates that 30% of women at some point in their lifetime experience violence with consequences of poor mental or physical health including, but not limited to, sexual and reproductive health problems, elevated risk of sexually transmitted diseases, depression, anxiety, self-harm, misuse of harmful substances, personality disorder and post-traumatic stress disorder (1–3). VAWG is a violation of women’s human rights and child sexual exploitation (CSE) is a significant component of VAWG.

CSE remains one of the most complex and challenging forms of VAWG and child abuse in our generation (4). Defined by the UK Government as a form of sexual abuse that occurs when “an individual or group takes advantage of an imbalance of power to coerce, manipulate or deceive a child… into sexual activity” either “in exchange for something the victim needs or wants”, or for the financial or reputational gain of the perpetrator (5), p. 5—CSE is now widely understood as rooted in coercion and exploitation. However, growing concern has emerged that this definition, with its emphasis on “exchange,” fails to fully capture the dynamics of grooming and control exercised by perpetrators. Scholars and practitioners alike have also warned that this definition risks obscuring the absence of meaningful choice or agency, especially in cases where victims are groomed, manipulated, and psychologically dominated and controlled into compliance (3, 6, 7).

In addition to the broader harms associated with VAWG, CSE carries further specific consequences for young people. Victims may experience disrupted adolescent development, cognitive dissonance regarding their relationships with perpetrators, and involvement in offending under coercion or manipulation (3, 8). Feelings of shame and self-blame are common, often compounded by societal judgement and policing interactions, which can limit victims’ ability to recognise their exploitation and seek help (9).

Children affected by CSE frequently experience overlapping vulnerabilities that intensify their risk for CSE and complicate service responses. These include previous abuse, care experience, family instability, and economic hardship (10). While girls are particularly overrepresented in CSE statistics and are disproportionately subjected to gendered stereotypes and victim-blaming attitudes (10) particularly with older victims (11), p. 13 boys and gender diverse children are also at risk, underscoring the need for inclusive responses. Although legal and professional frameworks assert that no child can consent to their own exploitation, victims are still often seen as complicit by perpetrators, professionals, and sometimes the public (3). The testimony of a young girl cited by Jay and others (3), p. 8 highlights this issue starkly: *“I was told by [care home] staff that I was attention seeking… crying for help… but no one ever paid attention. I was just treated like I was disgusting for doing it, not that there was a reason behind me doing it.”*

### 1.1 Policing responses to girls with experience of CSE

Tension around credibility, consent, and victimhood is particularly visible in policing responses to CSE (12, 13). Societal and institutional narratives continue to favour the ‘ideal victim’, perceived as passive, innocent, and blameless, while children with prior vulnerabilities, criminal records, or histories of running away are often seen as less credible or “troublesome” (14). This binary framing masks the complex reality that some children who experience CSE are also involved in offending behaviour, occupying what has been described as a victim-offender or dual-status position, often as a result of coercion, survival strategies, or grooming by perpetrators. Emerging empirical evidence demonstrates strong associations between childhood sexual abuse victimisation and adolescent offending, highlighting how exploitation and criminalisation frequently intersect rather than operate as separate trajectories (15). Further complicating matters, many children do not recognise their experiences as exploitation while they are happening which undermines both self-identification and willingness to engage with services (16). These dynamics pose significant challenges for investigation, prosecution, and support (17).

Despite some improvements in policy and safeguarding procedures, frontline responses to CSE, especially police interactions with girls, remain inconsistent, and at times harmful. Young people report feeling disbelieved, judged, or treated more as offenders than as victims (18). As one young person recalled of their experience with the police: *“One officer told me I was what was going wrong in our society… Another one said we were going to get these men in trouble because we wanted to act like child prostitutes”* (3), p. 8. Problematic victim-blaming attitudes among professionals mandated to protect children from harm are not uncommon. Further to this, even well-intentioned investigative procedures laid out in the Achieving Best Evidence guidelines for Video Recorded Interviews risks re-traumatising victims if officers lack the skills to recognise or respond to signs of trauma (13, 19, 20). Evidence also suggests that unconscious bias, which is often linked to child’s age, gender, immigration status, race, or class, can shape how children are perceived and treated by professionals (8, 12).

These shortcomings in criminal justice responses to CSE are reflected in consistently low conviction rates, particularly for sexual offences perpetrated against children. While there is considerable public expectation that reports of sexual abuse will lead to justice, high attrition rates remain a defining feature of these cases (21). Prosecutions frequently fail due to evidential issues, retracted statements, victim non-engagement, or difficulty establishing non-consent; especially where exploitation involves grooming or manipulation rather than physical force (17, 22, 23). Victims may withdraw from the process because of fear, shame, perceived disbelief, or lack of confidence in the system (12, 18, 24). A meta-analysis of 126 studies by Allnock et al. (25) found that poor inter-agency communication, limited confidence in talking with children about abuse, and an absence of evidence-led prosecution strategies were key barriers to justice; particularly when victims are unable or unwilling to participate in the criminal justice process.

### 1.2 Trauma informed approach

In response, trauma-informed approaches (TIA) have gained increasing traction as a framework for improving policing practice. Drawing on the principles of safety, trust, choice, collaboration, empowerment, and cultural awareness (26–28), trauma-informed policing recognises the enduring impact of trauma on victims’ behaviour and presentation. As a first in developing trauma informed investigative practices, officers are encouraged to ask “what happened to you?” instead of asking “what is wrong with you?”; a reframing that helps situate seemingly ‘difficult’ behaviours within a context of trauma and survival (29). A TIA does not require officers to become therapists or trauma specialists, but rather to develop a working understanding of how trauma manifests in people affected by traumatic experiences and how police behaviour during interviews, evidence-gathering, or routine engagement, can either escalate or de-escalate distress (13, 19, 20). Research suggests that trauma-informed policing practice can enhance victim engagement, reduce attrition, and contribute to both victims and officer wellbeing (19, 30).

Additionally, TIA in policing are not limited to victims of crime but are increasingly recognised as relevant when engaging with people who offend, particularly where offending is shaped by prior trauma, adversity, or coercion. McLachlan (31) argues that trauma-informed criminal justice requires attention to the lived experiences of those who offend, challenging punitive responses that may inadvertently exacerbate harm and re-entrench cycles of criminalisation. This perspective is especially pertinent in the context of CSE, where children and young people may occupy dual positions as victims and offenders, and where TIA must navigate complex intersections of vulnerability, responsibility, and safeguarding. Embedding this broader understanding within policing practice supports more proportionate, humane, and effective engagement across investigative and enforcement contexts.

Some of the challenges and barriers to seeking justice for CSE victims and survivors are compounded when children do not realise they have been exploited or feel responsible for what has happened to them. Even when children recognise their exploitation, engagement with the criminal justice process itself can also be a source of considerable distress. McElvaney and others (32), drawing on semi-structured interviews with 47 young people aged 14–25 across Ireland and Canada, highlight the importance of feeling safe through kindness, transparency, and being believed, as well as having a voice in proceedings. However, victim-blaming narratives remain common, with sexually exploited children often perceived as making a ‘life choice’, diverting attention away from the perpetrator (33). Understanding the young person’s perspective and interpreting behavioural manifestations as indicators of trauma rather than defiance or consent is a crucial starting point for police officers investigating CSE offences (33).

Part of a TIA includes looking at the impact of secondary trauma on officers. Secondary trauma among law enforcement officers is a well-documented occupational hazard, particularly for those working with sexual violence and child exploitation cases. Studies show that repeated exposure to traumatised individuals, distressing materials, and emotionally charged investigations can contribute to compassion fatigue, burnout, and secondary traumatic stress (34, 35). Factors such as role overload, organisational pressures, and lack of recognition can compound these effects, while gender, length of service, and type of crime investigated may influence vulnerability (36, 37). Negative outcomes can extend to anxiety, depression, and disruptions in personal relationships, particularly when officers experience moral injury or institutional betrayal (38, 39). Evidence suggests that multi-level support systems, combined with trauma-informed approaches, can improve officer wellbeing, retention, and the quality of victim engagement (40).

### 1.3 Experiential training

Though a TIA to policing is becoming widely acknowledged, embedding this way of working in policing practice requires more than knowledge transfer. While awareness-raising is necessary, traditional classroom-based training is often insufficient for developing the emotional literacy, relational sensitivity, and reflective capacity that trauma-informed work demands. Experiential learning models offer an alternative, grounded in the idea that learning is more effective when it is embodied, relational, and contextualised. As Gitterman (41) noted, much of professional competence is “caught” rather than “taught,” and Sheppard (42) argued for “practice validity” in training to build authentic, transferable practice wisdom. Simulation-based learning, in particular, enables immersive engagement with high-stakes, real-world scenarios; allowing for trial, error, feedback, and reflection in a psychologically safe environment (43–45).

Simulation based learning and teaching has been widely adopted across professional sectors, including the military, emergency services, healthcare, and policing, as a means of preparing individuals for complex, unpredictable situations (46). Crucially, evidence also suggests that interactive, experiential interventions are more effective than didactic forms in disrupting entrenched cognitive biases, with impacts that can persist months after training (47). Within the context of CSE, simulation-based learning offers a unique opportunity to prepare officers for the nuanced, emotionally charged realities of working with traumatised young people. It brings abstract training principles to life; requiring officers to recognise trauma responses in real time, navigate emotional tension, challenge their own assumptions, and develop meaningful relational approaches.

Despite growing recognition of the importance of trauma-informed approaches within policing, the empirical evidence base examining how such approaches are developed, operationalised, and embedded in police practice remains limited (48). In particular, there is a paucity of research exploring co-design methodologies in the development of trauma-informed training interventions, especially those that meaningfully involve collaboration between academic researchers and frontline practitioners (49). While simulation-based and experiential learning models are increasingly advocated as promising mechanisms for supporting trauma-informed practice, few studies have examined their development and evaluation through participatory or action-oriented frameworks. As a result, there is limited understanding of how co-produced training tools may support both cultural and practice change within policing responses to child sexual exploitation.

This article presents findings from a co-designed, simulation-based training intervention developed through participatory action research framework to support police officers in applying TIA when working with girls who have experienced CSE. Co-designed by academic and practice partners and grounded in research literature, the simulation aims to bridge the gap between theoretical principle and practice. It offers officers the chance to rehearse and reflect on their responses in complex interview settings, build relational competence, and challenge their own and others’ conscious and unconscious biases. The following sections outline the training’s development, implementation, and evaluation which highlights its potential to contribute to a more humane, effective, and evidence-informed police response to CSE.

## 2 Materials and methods

This project adopted a transformative participatory action research approach, underpinned by principles of co-production between academia and frontline professionals, with a focus on social transformation in police practice (50). Transformative participatory action research was chosen for its capacity to enable collaborative knowledge production, iterative learning, and actionable change within organisational contexts (50, 51). This approach aligns with the study’s critical constructivist epistemology, recognising that knowledge is co-constructed between researchers and practitioners and is shaped by power dynamics and contextual factors. Transformative participatory action research was particularly suited to our study, as it allowed iterative co-design of the simulation tool, integration of officers’ insights into training development, and real-time evaluation of its impact on trauma-informed practice. By embedding research within practical action and fostering stakeholder ownership, this approach ensured the study could both develop a relevant, usable training intervention and generate evidence about its effectiveness in enhancing officers’ skills and knowledge.

The study aimed to develop, implement, and evaluate a pilot simulation training tool, with evaluation activities drawing on mixed-methods design, to support police engagement with girls with lived experience of child sexual exploitation (GLE-CSE) in a trauma-informed manner. Participatory, action-oriented research and innovation form a cornerstone of effective simulation development in safeguarding contexts. Collaborative approaches bring vital frontline expertise, lived experience, and access to professional datasets, all of which are essential for relevant and impactful design and application (50). This approach was chosen to foster meaningful professional learning and cultivate stakeholder ownership of the final training intervention (50).

The project was guided from the start by the following research questions:

1. What key features should be included in a pilot simulated training tool for police to foster a trauma informed approach to support, help and intervention with GLE-CSE?
2. What do police delegates value, dislike or desire from the pilot training tool created to promote a trauma informed approach to support, help and intervention with GLE-CSE?
3. How do police feel the pilot simulation training tool has impacted on their knowledge, skills, and behaviours around trauma informed approaches to support, help and intervention with GLE-CSE?

These research questions were addressed across three distinct and structured project phases between November 2022 and October 2023. These phases included the collaborative co-design of the pilot simulation tool; the development and production of the pilot simulation tool; and the delivery and evaluation of the training using the pilot simulation tool with frontline police officers. These phases will be reviewed in turn below.

### 2.1 Phase 1: collaborative co-design of the pilot simulation tool

In addressing research question 1, the research team initiated a co-design phase involving structured collaboration between the Centre for Child Protection (CCP) at the University of Kent and Kent Police. A series of planning workshops took place between November 2022 and February 2023 which were designed to foster an effective information exchange between academic and policing stakeholders. This included relationship building, exploration of existing case studies, the sharing of unique professional datasets, reviewing published/unpublished research, consideration of the voice of a young person who has been a victim of CSE in an anonymised fashion to inform Kent Police practices, the exchange and sharing of subject specific academic literature (child sexual exploitation, trauma informed practice, and simulation based learning), and frontline policing practices. The project team also consulted Kent Police’s Equality, Diversity and Inclusivity Team in the creation of the main characters for the pilot simulation to ensure suitability and thoughtful representation. In the latter part of this iterative knowledge exchange, key learning outcomes were agreed alongside the development of a detailed training blueprint which included:

- Structure and storyline for an interactive pilot simulation
- Diverse character backgrounds and narrative perspectives
- Draft scripts and training strategies
- Interactive gaming element ideas
- Discussion points to facilitate small group discussion and shared learning

### 2.2 Phase 2: development and production of the pilot simulation tool

Utilising the outputs from the co-design phase, a pilot simulation training tool was developed using Articulate (52) software between March and June 2023. Alongside the construction of the digital pilot simulation, research question 1 was further expanded on during this phase. Additional supporting resources were created which drew from the initial knowledge exchange and were refined through ongoing collaborative check-ins between CCP and Kent Police. These iterative discussions were used to interrogate, validate, and further develop design choices to ensure fidelity to trauma-informed principles.

Additional elements integrated into the pilot simulation tool included:

- Printable worksheets for reflection and group work
- A facilitator training pack to support consistent delivery
- Academic literature review synthesising research on policing practice with GLE-CSE and trauma-informed approaches

This phase ensured that the training package was both practically usable and theoretically grounded, enabling frontline officers to engage with key concepts in a structured and reflective manner. The pilot training programme, called Robyn and Molly, was ready for delivery and evaluation.

### 2.3 Phase 3: delivery and evaluation of the pilot simulation tool

To answer research questions 2 and 3, the pilot simulation tool was implemented with a cohort of 77 Kent Police officers across nine sessions between July 2023 and October 2023. A purposive, criterion-based sampling strategy was employed to recruit participants with direct professional relevance to the aims of the pilot training intervention. Participants were identified by Kent Police internal leads and selected based on their active involvement in cases involving GLE-CSE or if they were members of child protection units. This approach reflects the objectives of the pilot participatory action research study, where sample size is determined pragmatically and is intended to support feasibility testing, professional learning, and rich qualitative feedback rather than statistical generalisability.

Training sessions were facilitated jointly by CCP and Kent Police, drawing on the training experience of both organisations. Each training session included live facilitation, structured engagement with the pilot simulation tool, and large and small group discussion-based activities. Sessions lasted for 1 full day (10am–4pm, with a lunch and short breaks) and were delivered in small groups of 11 or under to maximise reflective interaction among delegates.

Participants were informed that their anonymised feedback would be used to evaluate and improve the pilot simulation tool. Attendance and participation were voluntary. However, in the spirit of participatory action research, delegate feedback was incorporated into the pilot training simulation; further shaping the training tool for suitability of purpose and improved impact through iterative cycles of reflection and refinement.

#### 2.3.1 Data collection and evaluation methods

Pilot training evaluation was conducted using a mixed-methods approach to assess impact across knowledge, skills, and practice domains. Data collection included pre-, mid-, and post-training questionnaires distributed via an online survey link, alongside a live polling activity delivered through Mentimeter (53). These tools captured participant feedback on the pilot simulation training and provided space for self-assessment against the intended learning outcomes. Both formats included Likert-scale items (e.g., confidence in using trauma-informed approaches, understanding of GLE-CSE) and open-text responses (e.g., reflections on what was most or least valuable). Additionally, each session was independently observed by someone external to the training team, providing contextual insight that enriched the interpretation of participant feedback and grounded findings in the realities of frontline delivery.

#### 2.3.2 Data analysis

Quantitative data from Likert-style responses were analysed using descriptive statistics (54) to identify trends in participant confidence, knowledge acquisition, and self-reported changes in practice. Qualitative data, including open-text responses and independent observations, were thematically analysed (54) using an inductive coding approach, allowing for key themes to emerge directly from the data.

### 2.4 Ethics

Ethical approval was granted by the University of Kent’s Ethical Staff Review Committee (Ref: 0713) in October 2022.

## 3 Results

Gains were notable among trainee detectives, though experienced officers from child exploitation, missing persons, and child protection teams also rated the training as relevant and valuable. This section addresses the study’s three research questions, beginning with the key features of the pilot simulation training tool, followed by participant perceptions, and finally the impact on knowledge, skills, and behaviours.

### 3.1 Key features of the pilot simulation training tool

Phases 1 and 2 produced six learning objectives (LO) for the Robyn and Molly simulation:

1. Develop knowledge around identification and recognition of CSE
2. Understand how to support girls with lived experience of CSE with a trauma informed approach
3. Develop skills around active investigations of CSE
4. Understand how to prepare and present CSE cases to the Crown Prosecution Service
5. Recognise and respond to secondary trauma in oneself and with colleagues
6. Develop skills to engage with girls who have lived experience of CSE

**Figure 1:**
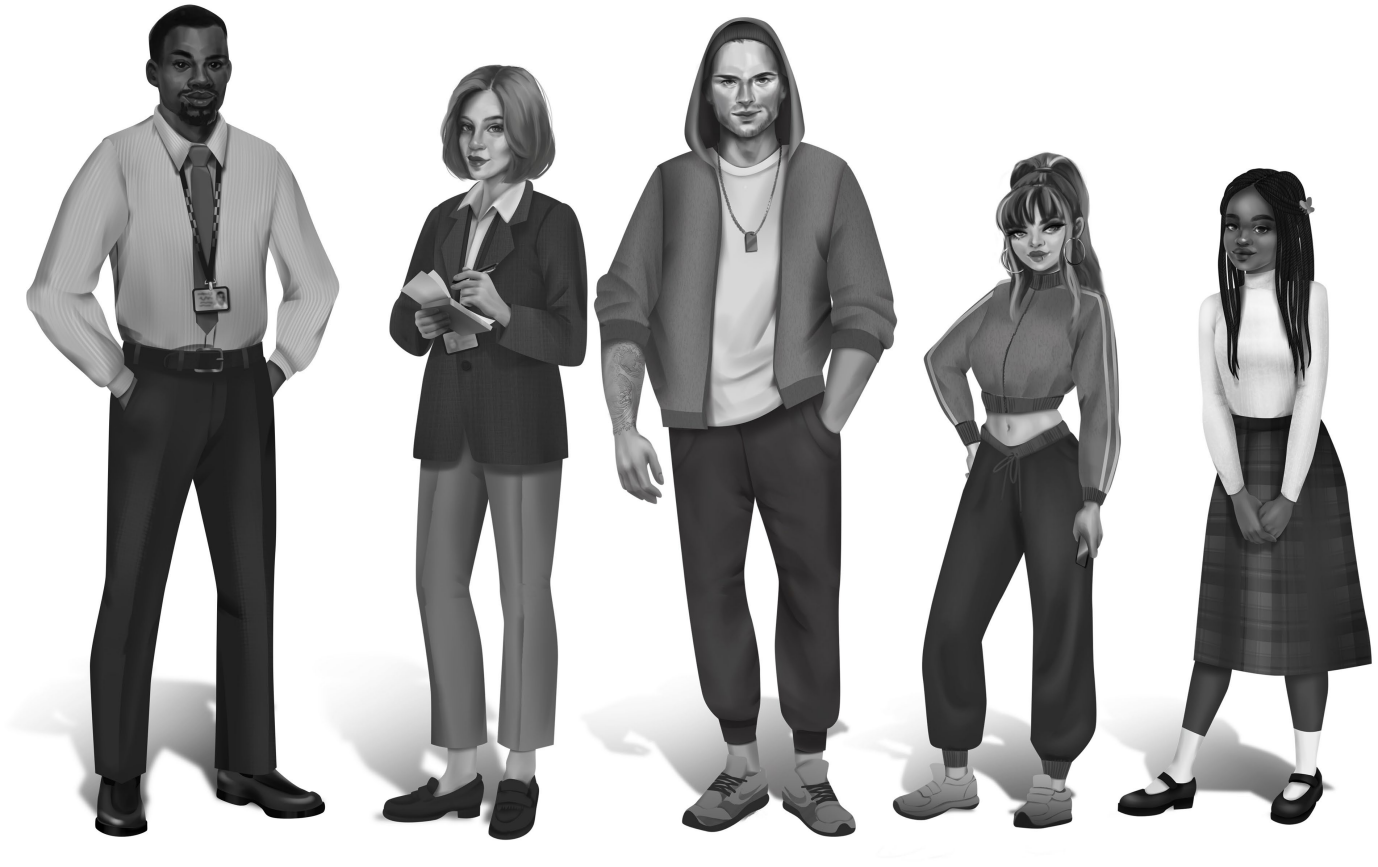
Sketches of main characters in Robyn and Molly simulation training tool.

**Figure 2:**
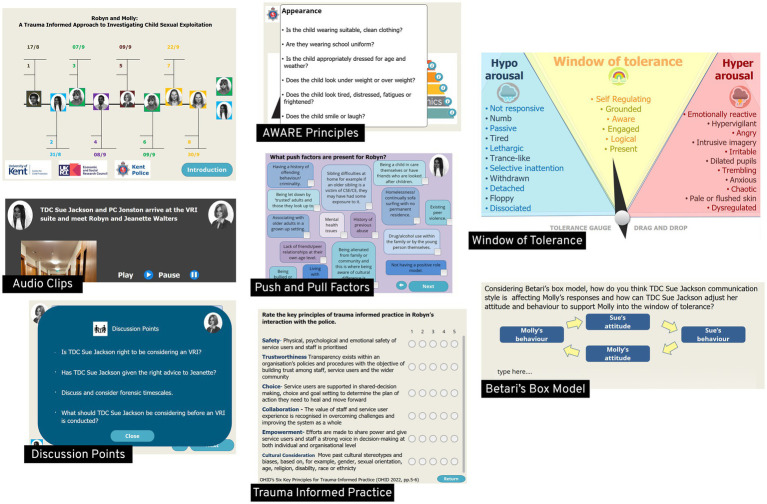
Interactive gaming activities and discussion points in the Robyn and Molly simulation.

These first phases also saw visual sketches developed by Playerthree (digital production partner; Figure 1) and the agreement of interactive activities and discussion points embedded throughout the training tool. These activities and discussion points were carefully developed and embedded within the programme design to support acquisition of above LOs. A timeline navigation tool incorporated interactive content, including the AWARE principles mnemonic, which guided officers to consider appearance, words, activities, relationships (and dynamics), and the environment when engaging with young people. A complementary “window of tolerance” gauge was designed to support officers in recognising hypo- and hyper-aroused states in young people, family members, and themselves, highlighting how these states can affect evidence gathering and risk of traumatisation while considering strategies to restore regulation. Additional activities encouraged officers to listen to and assess key scenes, explore push and pull factors associated with CSE, and engage with thought-provoking discussion points in both small- and large-group settings. The programme also incorporated ongoing assessment of trauma-informed principles across interactions and applied concepts such as Betari’s Box to deepen understanding of the influence of self and the perspective of others (see Figure 2). Betari’s Box illustrates how an individual’s attitudes shape their behaviour, which in turn affects the attitudes and behaviours of others, creating either constructive or destructive cycles of interaction.

### 3.2 Participant perception of the pilot tool

Evaluation questionnaires were completed by all participants (*n* = 77). Most participants (94.8%, 73/77) rated the training as relevant or highly relevant; three found it irrelevant, although two of these still described the content as useful but more suited to new recruits. Almost all participants (98.7%, 76/77) reported enhanced knowledge and understanding of trauma-informed approaches to CSE investigations. The single dissenting participant felt the content repeated existing knowledge. The majority (89.6%, 69/77) agreed that the course met expectations; qualitative comments suggested that uncertainty among others was often due to a lack of pre-course expectations rather than dissatisfaction.

What participants valued most included interactive tool design, group discussions, and audio elements. They valued the mixed delivery methods and knowledgeable trainers as well as comprehensive resource packs and a safe space for reflection. Suggestions for improvement clustered around tool design, content and delivery. For tool design, delegates wanted clearer audio, more immersive visuals, improved navigation (e.g., back buttons, progress bar), integrated notetaking, richer character backstories, and enhanced accessibility. Regarding content, there were comments about wanting greater emphasis on trauma-informed science, child development, rapport building, court preparation, and further insight into children’s lived-experience through quotes/voices of children and less focus on Achieving Best Evidence procedures. Finally, the delivery preference was for longer duration (two days) alongside more group work, formal tool introduction, improved time management, and fewer written tasks.

Several suggestions were implemented in subsequent deliveries, including improved navigation, streamlined discussion points, richer character backgrounds, reduced focus on Achieving Best Evidence procedures, additional trauma science, and Crown Prosecution Services (CPS) preparation content. Recommendation for two-day delivery remains under review due to logistical constraints. This remains an area for consideration; however, we found the 1-day delivery worked well with revisions towards the end of training resulting in similar concerns not being raised as much.

### 3.3 Impact on knowledge, skills and behaviours

#### 3.3.1 Learning objectives

**Figure 3:**
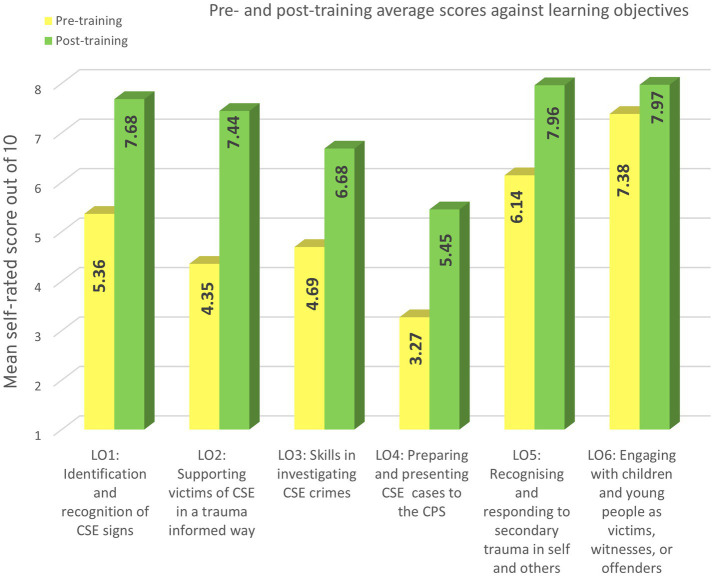
Pre- and post-training average scores against learning objectives.

Figure 3 presents mean pre- and post-training self-ratings against the six learning objectives. All areas showed improvement which is discussed more.

##### 3.3.1.1 Increased knowledge in recognising CSE signs (LO1)

Identifying signs and indicators of CSE had mean scores increased by 2.32 points (+53.2%), with the proportion scoring ≥7 rising from 36% pre-training to 85% post-training.

##### 3.3.1.2 Trauma-informed victim support (LO2)

Supporting victims of CSE in a trauma-informed way showed the greatest improvement, with mean scores rising by 3.09 points (+71%). The proportion scoring ≥7 increased from 18% to 78%.

##### 3.3.1.3 Skills in investigating CSE crimes (LO3)

Perceived CSE investigative skill’s mean scores improved by 2 points (+42%), with the proportion scoring ≥7 increasing from 19% to 49%.

##### 3.3.1.4 Case preparation and presentation to the CPS (LO4)

Considering preparing and presenting CSE cases to the CPS identified a significant baseline knowledge gap, with 49% scoring ≤2 pre-training. Following training, the proportion scoring ≥7 rose from 8% to 42% with the overall mean score raising 2.18 points (+67%).

##### 3.3.1.5 Responding to secondary trauma (LO5)

Confidence in recognising and responding to secondary trauma in self and colleagues increased by 1.82 points (+30%), with 86% scoring 7 or above post-training (compared to 74% pre-training). It was good to see this was one of the higher baselines; and even better to see such a significant improvement in this area, too.

##### 3.3.1.6 Engaging children and young people (LO6)

Baseline confidence in engaging with children and young people as victims, witnesses, or offenders was relatively high (mean = 7.38). Post-training improvement was modest with a 0.59 mean increase (+8%); however, the proportion scoring 7 or above still rose from 59% to 68%, indicating positive reinforcement even in high-confidence areas.

#### 3.3.2 Impact

Post-training review identified improved recognition and recording of trauma in police files. Investigators demonstrated a significant shift in understanding the impact of past trauma on young people’s presenting behaviours. Between 03/2023–05/2023 (pre-training) and 10/2023–12/2023 (post-training), the presence of the word *trauma* in case assessments raised 133%. Importantly, this occurred alongside a regional decrease in missing person reports, suggesting more accurate and sensitive assessments of vulnerability rather than changes in case volume. Case notes showed tangible improvements, with victim-blaming language replaced by trauma-informed observations, e.g., describing young people as “struggling with emotional regulation due to adverse childhood experiences.”

Kent Police also reflected on practical and cultural benefits post training. Delegates reported that training directly supported their engagement with children, including Achieving Best Evidence interviews, risk assessments, and investigative planning. Kent Police highlighted the training’s organisational impact, recognising it as an “innovative solution” to challenges in large-scale staff training, and noted its role in fostering a victim-centred culture by reducing victim-blaming language and embedding trauma-informed principles. The pilot training has informed discussions about making the simulation mandatory and has attracted interest from other policing forces, highlighting its potential for broader dissemination.

## 4 Discussion

This study provides one of the first empirical evaluations of an immersive simulation-based training tool specifically designed to embed trauma-informed approaches within police responses to girls with lived experience of child sexual exploitation (GLE-CSE). Building on over a decade of the Centre for Child Protection’s research-informed simulation development, the pilot was delivered in partnership with Kent Police (KP) and targeted the identified gap in specialist CSE training for investigators (3, 18, 25). The innovation lies in translating complex theoretical concepts, such as the neurobiological impact of trauma, the cyclical nature of exploitation, and the role of shame, into experiential, practice-based learning components.

The training directly addressed persistent issues highlighted in national reviews, including inconsistent recognition of CSE indicators, the persistence of victim-blaming attitudes, and variable application of trauma-informed practice (55, 56). Unlike traditional classroom formats, the immersive simulation allowed officers to “experience” victim interactions, receive real-time feedback, and rehearse language and behaviours aligned with trauma-informed principles. This approach resonates with pedagogical evidence that interactive, scenario-based learning is more effective at embedding complex relational skills than didactic training alone (57, 58).

### 4.1 Increasing recognition of trauma

The most substantial gains were observed in officers’ ability to identify signs and indicators of CSE and apply trauma-informed responses. Mean scores for recognising CSE indicators increased by over 50%, while the proportion of officers referencing trauma in case notes more than doubled in the months following training. These changes suggest that the training not only increased knowledge but also shifted investigative framing, with officers more likely to interpret victims’ behaviours (i.e. going missing or disengaging from services) through a trauma lens rather than as wilful non-compliance.

This is a critical development given research showing that CSE victims often present with complex behaviours that can mask their victimhood (59, 60). Misinterpretation of such behaviours can perpetuate victim-blaming and undermine both safeguarding and evidential processes (61). The post-training removal of victim-blaming language from case notes, replaced with references to adverse childhood experiences and emotional regulation difficulties, represents a tangible cultural shift within investigative practice. Such linguistic changes are not superficial; they have been shown to influence decision-making, inter-agency collaboration, and victims’ willingness to engage with the justice process (62).

These gains are also relevant for children who may occupy dual roles as victims and offenders. Trauma-informed policing encourages officers to interpret offending behaviour in the context of prior victimisation, coercion, or survival strategies, rather than as wilful misconduct alone (15, 31). In practice, this means officers are more likely to recognise trauma in victims’ behaviours, but also to approach instances of offending with proportionate, context-sensitive responses; balancing safeguarding, accountability, and support. Embedding this perspective helps prevent re-traumatisation, reduces punitive responses that may exacerbate harm, and promotes engagement with both victims and those involved in harmful behaviours.

### 4.2 Reduction in victim-blaming language

Victim-blaming narratives which frame young people as making “choices” to be exploited, have been widely criticised for obscuring the coercive and grooming processes underpinning CSE (3, 13, 63). The finding that these narratives diminished in case documentation after training aligns with evidence that targeted and experiential professional development can disrupt entrenched cognitive biases (47). By embedding trauma-informed approaches, the training appears to have provided officers with both the conceptual understanding and the practical vocabulary to reframe victims’ behaviours. This reframing is likely to support improved rapport, facilitate more effective Achieving Best Evidence interviews, and strengthen case preparation for the Crown Prosecution Service (CPS).

### 4.3 Investigative and case preparation skills

While the greatest post-training gains were seen in recognising CSE and embedding trauma-informed approaches, improvements were also evident in investigative practice and case preparation for the CPS. Pre-training data revealed significant gaps in officers’ knowledge of case-building processes, with nearly half scoring at the lowest levels for this learning outcome. Post-training, the proportion achieving high scores more than quadrupled. This is particularly relevant given longstanding concerns about low prosecution and conviction rates in CSE cases, often linked to evidential weaknesses and missed opportunities to corroborate victim testimony (17, 21–23).

By engaging with realistic case scenarios, participants were able to rehearse investigative decision-making, consider evidential strategies, and appreciate the cumulative impact of early victim engagement on case outcomes. The integration of trauma-informed practice into investigative processes is significant; national guidance increasingly emphasises that a victim’s willingness to engage and provide coherent accounts is shaped by how they are treated in the early stages of an investigation (12, 18, 25). The simulation therefore addressed both the relational and procedural dimensions of effective CSE policing, an area often treated separately in traditional training formats.

### 4.4 Practical implications

The findings highlight the potential for simulation-based training to achieve measurable improvements in both knowledge and cultural attitudes within policing. Importantly, the observed changes were sustained beyond the immediate post-training period, with officers reporting 3–6 months later that they had applied learning directly in live cases, including adapting interview techniques, seeking intermediary support for Achieving Beset Evidence interviews, and reframing risk assessments. Such sustained application suggests the training was not merely a one-off knowledge boost but contributed to changes in professional identity and practice norms.

The increase in recorded references to trauma in case notes despite an overall regional decline in missing person reports, provides additional evidence of cultural and procedural change. These findings are consistent with wider research on simulation and experiential learning, which shows that embodied, scenario-based experiences can lead to longer-term skill retention and transfer to practice (41–45, 47). For policing, where time pressures and competing priorities can undermine training effectiveness, the immersive format offers an efficient means of embedding complex knowledge into operational practice.

### 4.5 Organisational and cultural change

One of the most notable outcomes of the pilot was the shift in organisational language and framing of CSE victims. Feedback from Kent Police emphasised that the training supported a more victim-centred culture and helped to “eradicate victim-blaming language.” This is an important finding given that culture change within policing is often slow to achieve and resistant to short-term interventions (64). The fact that senior leaders identified the simulation as offering “innovative solutions” and improving service to “our most vulnerable young victims” suggests institutional buy-in, which is essential for sustainable change.

The potential scalability of the pilot is also significant. Interest from other policing forces indicates recognition that the training addresses a critical skills gap across forces, aligning with the Home Office’s *Tackling Child Sexual Abuse Strategy* (65) and the broader Violence Against Women and Girls (VAWG) agenda. Embedding this training at a national level could contribute to standardising trauma-informed practice in CSE investigations, reducing variability between forces, and improving both safeguarding and criminal justice outcomes for victims.

### 4.6 Secondary trauma and implications for practice

While the primary focus of this study was on improving police engagement with victims of CSE through trauma-informed approaches, it is important to acknowledge the parallel impact of this work on officers themselves. Secondary trauma, arising from repeated exposure to distressing narratives, materials, and interactions, is a well-documented occupational hazard in child sexual exploitation investigations (34, 35). Left unaddressed, this can contribute to burnout, moral injury, and reduced capacity for empathic engagement.

The simulation deliberately incorporated reflective opportunities to address this challenge. For example, one scene follows a trainee detective who becomes emotionally overwhelmed by a case and seeks supervision with her line manager. In this interaction, the detective is supported to process her experiences, consider her role within the complexity of her relationship with a girl with lived experience of CSE, and reframe her understanding of the young person’s behaviour through a trauma lens. This allowed her to see how past adversities have shaped her current situation rather than resorting to victim-blaming interpretations. Such embedded reflection models good supervisory practice, reinforces the value of emotional processing in sustaining effective engagement, and underscores the need for organisational structures that actively support officer wellbeing.

### 4.7 Limitations and future research

This pilot study has several limitations that should be acknowledged. First, while pre/post measures indicated statistically and practically significant improvements across all learning outcomes, the reliance on self-reported confidence and knowledge introduces the possibility of response bias (66). Objective measures of behavioural change in live investigative contexts such as formal case audits or third-party observational data, would strengthen the evidence base. Second, the sample was limited to investigators from a single police force. While Kent Police is broadly representative in its CSE caseload, differences in force culture, resources, and local partnership arrangements may influence training outcomes.

The follow-up qualitative feedback collected 3–6 months post-training is encouraging, suggesting sustained application of learning; however, longer-term tracking is required to determine the durability of changes and their impact on case outcomes. Future research could explore comparative trials across multiple forces, incorporate victim feedback on officer interactions post-training, and evaluate whether improved investigative practice translates into increased charging and conviction rates in CSE cases. Additionally, further study into how simulation design features such as narrative complexity, character diversity, interactive features, affect learning transfer could optimise future training development.

## 5 Conclusion

This study demonstrates the potential of immersive, simulation-based training to embed trauma-informed approaches in CSE investigations and improve both procedural and relational policing skills. The findings indicate significant gains in officers’ ability to recognise indicators of CSE, apply trauma-informed principles, prepare robust cases for the prosecution, and reduce victim-blaming language in practice. The increase in recorded consideration of trauma, alongside reductions in missing person reports, points to a tangible shift in both investigative framing and organisational culture.

By integrating realistic scenarios with evidence-based pedagogy, the pilot-simulation translated complex theoretical concepts into operational competence, bridging a longstanding gap in traditional police training. The positive reception from both practitioners and senior leadership, combined with interest from other policing forces, underscores the potential for scaling this approach. These findings should, however, be interpreted with appropriate caution. The study relied primarily on self-reported measures of knowledge, confidence, and perceived practice change, which may be subject to response bias and cannot, on their own, confirm sustained behavioural change or impact on investigative outcomes. In addition, the contextual specificity of a single-force pilot limits the generalisability of findings across different organisational cultures, resources, and policing contexts. Nevertheless, as an embedded participatory evaluation, the study provides robust practice-based evidence of feasibility, acceptability, and perceived impact, offering a strong foundation for future multi-site and longitudinal research aimed at strengthening trauma-informed responses to CSE.

## Data Availability

The datasets presented in this study can be found in online repositories. The names of the repository/repositories and accession number(s) can be found at: https://data.kent.ac.uk/510/. Please note that the deposited materials comprise only non-identifiable project outputs (simulation presentation, blank evaluation forms, and character sketches). No participant-level or sensitive data are shared. Access is provided for transparency and replication purposes.
